# Neighborhood Effects, Mental Illness and Criminal Behavior: A Review

**DOI:** 10.5539/jpl.v6n3p1

**Published:** 2013-08-30

**Authors:** David Freedman, George W. Woods

**Affiliations:** 1 Department of Epidemiology, Mailman School of Public Health, Columbia University, New York, NY, USA; 2 Morehouse University School of Medicine, Atlanta, GA, USA

**Keywords:** death penalty, neighborhood effects, race, poverty, criminal behavior, mental illness

## Abstract

This paper briefly reviews the social science on “neighborhood effects” as an independent force in shaping poor outcomes, specifically mental illness and criminal behavior, before discussing the implications of that research for understanding the relationship between neighborhoods, race and class. Neighborhood effects research has proliferated in recent years with extensive attention again being focused on the social context of family and individual development and life course. Moreover, recent work has suggested the need to consider the developmental effects of neighborhoods that persist across life-span. This paper will focus specifically on mental illness and criminal behavior as outcomes for understanding neighborhood effects, but will also consider what the structural causes of individual behavior and functioning mean for clinical assessment, especially forensic assessment.

## 1. Introduction

Neighborhood effects research, using an expansive array of data and analyses, has made significant strides in the last twenty-five years (Raudenbush & Sampson, 1999). The relationship between neighborhoods and poor mental and physical health, although studied for decades (Faris, 1939, Reprinted 1965), now more clearly and strongly links neighborhoods to mortality, heart disease, cancer, low birth weight, infant mortality, childhood illnesses, asthma, depression, anxiety, smoking, diet and nutrition, hypertension, heart disease, suicide, accidental injuries, lead exposure, and numerous other illnesses (Roux & Mair, 2010; Morenoff & Lynch, 2004). Research on the association of family and neighborhood characteristics with delinquency and crime also began more than seventy-five years ago, reaching conclusions about the fundamental role of family life and neighborhood in behavioral problems that have been confirmed over the decades since (Glueck & Glueck, 1950; Healy & Bronner, 1936; Sampson & Laub, 1993; Shaw & McKay, 1969). Nevertheless, serious research issues remain concerning both how to measure neighborhood effects and how to interpret results that suggest associations (Roux, 2008; Oakes, 2004).

Recently, Sampson (2008) articulated an important conceptual framework for understanding the mechanisms by which neighborhoods effect individuals (Sampson, 2008). This framework argues for a dual import to neighborhoods: first, as the situational context of family and individual life - which has long been how neighborhoods are viewed (Bronfenbrenner, 1977); but second, as influencing the developmental and enduring early life course that shapes long-term development, behavior and health throughout the life of the individual regardless of subsequent neighborhood stability or individual mobility (Sampson, 2008). In considering outcomes such as mental illness and criminal behavior, this dual framework suggests important possibilities for understanding and preventing illness and crime, and therefore, is also important for clinical and forensic neuropsychiatric practice.

In this paper, we first briefly review some of the research related to neighborhoods and mental health in adults and children. We next focus more specifically on the research related to psychosis, child abuse and witnessing violence, neurotoxicant exposure and finally criminal behavior. As discussed throughout these sections, issues of race/ethnicity and poverty are interwoven into the research findings, but we also address more directly race and class and neighborhoods in considering the pathways and mechanisms by which neighborhoods may be associated with these outcomes.

## 2. Mental Illness and Neighborhoods

Historically, mental illness has been seen as a condition of the individual alone. Individuals, rather than families or communities, are diagnosed with mental illnesses, except in certain rare disorders, like shared delusional disorder, where several individuals, or even a community may suffer shared psychiatric symptoms. Certainly, few would disagree that mental illness is embodied in the individual. As a result, mental illness is susceptible to the individualistic fallacy which assumes that individual-level outcomes should be attributed solely to individual characteristics or traits (Silver, 2000). However, families and neighborhoods have clear associations with, and in some instances appear to be causally related to, mental illnesses. Current research finds independent, statistically significant effects when examining neighborhoods and mental illness. As Oakes writes: “It would be shocking to learn that [social] contexts did not somehow impact health. The question is about magnitude, mechanism, and mutability” (Oakes, 2004) at p.1929. The effect sizes are often small, leading to debate as to the significance of association. Yet, the question for people interested in identification, prevention, and treatment of mental illness and shifting the career course of offenders, as well as those interested in understanding the neurodevelopment and life course, is not to parse the exact contribution of one risk factor while holding all else constant, but rather to understand the interaction and mediation of associated risks that act together, both the direct and indirect effects of neighborhoods, to shape the life experiences of those who become and are mentally ill (Hafeman, 2008).

Further, while neighborhoods are the collection of individuals, suggesting that aggregated individual data might explain the neighborhood, they are also more than the sum of the individual parts. Neighborhoods have quantifiable characteristics above and beyond the aggregated individual level. For instance, vacancy rates in residential areas are a measure of the neighborhood characteristics, not the individuals who do or do not live in the area. Similarly, a neighborhood which is densely poor, may in fact have some individuals who are exceptionally wealthy. To simply use mean or median income as a measure of the neighborhood may misrepresent the lived experience of those in the neighborhood, thereby missing or mischaracterizing the effect of social context on that lived experience. Additionally, the rate of change over time of a neighborhood may be quite different than the duration of residence of any set of individuals in that area, reflecting qualities other than the aggregated sum of residents at any given point in time. Finally, neighborhoods may change by virtue of policy (changes in zoning, increased infrastructure investment to improve walkability, or the lifting of restrictive covenants) or by virtue of adjacent neighborhood changes (increased public transportation or expansion of services in the adjacent neighborhood), influences which have no individual level corresponding characteristic.

Neighborhoods, then, are more than an aggregate of individual level characteristics, but that does not sufficiently define what constitutes “neighborhood” or what should be measured to capture its characteristics. Most simply, neighborhood are the utilized space in the daily functioning of people and families, the place where people live and move to work or play or school, to shop, to interact with other people. Yet, such a simply definition would mean that every person has his or her own “neighborhood,” making the concept incoherent and quantitatively useless. Some researchers have used statistically available geographic boundaries (such as census tracts) or the aggregation of individual characteristics (the place where most people have a high socio-economic status). Some have proposed thorough approaches to defining neighborhoods for research purposes (Aronson, Wallis, O'Campo, & Schafer, 2007; Azrael et al., 2009; L. Weiss, Ompad, Galea, & Vlahov, 2007), but few studies utilize such careful and time intensive approaches. While our review is bounded by the definitions used by the researchers who conducted the studies, the conceptual approach to neighborhoods is predicated on the idea that neighborhoods are defined by physical space, shared social norms and expectations, social networks, and institutional structures.

Structural and distal causes of functioning and behavior affect groups and populations. Clinical and forensic practice often becomes over-focused on the individual, losing out on important influences and risks that increase the rates and risks for mental illness, as well as the possibilities of intervention and prevention. Thus, when we examine individuals and seek to understand why this person got this disease at this time, we are often unable to examine and understand the mechanisms by which an illness is distributed within a population (Rose, 1985). By understanding the role of neighborhoods and communities in mental illness, we are able to compare not just individuals to other individuals, but social contexts in which those individuals developed and lived and became ill.

### 2.1 Adults

Truong and Ma (2006) reviewed the literature on adult mental health and neighborhoods (Truong & Ma, 2006). They found 27/29 studies reported statistically significant neighborhood effect on mental health for adults. The studies used differing measures of neighborhood and outcomes, making comparisons difficult across the group of studies, but in general, the evidence supported the finding that neighborhoods have an independent effect on the incidence of mental illness, specifically: symptoms of depression, psychological distress, anxiety and psychosis. The studies fell into three groupings regarding the proposed mechanisms of these effects: structural characteristics of the neighborhoods (i.e., socio-demographic make-up), neighborhood disorder (i.e., perceived safety and social and/or physical “uncivility”), and environmental stressors (i.e., stressors and resources).

The United States Office of Housing and Urban Development (HUD) sought to directly test whether concentrated poverty caused worse mental health, employment, and school outcomes by conducting an experiment which randomized people into types of housing options. The Moving to Opportunity (MTO) project randomized a sample of people living in neighborhoods of concentrated poverty in five major cities, into three groups: the first group, a control group who continued to be eligible for public housing; the second group was referred to as the Section 8 group, received a Section 8 housing voucher without geographic restriction; and the third group, the experimental group, was given a Section 8 housing voucher that restricted the density of poverty in the census tract to which they were permitted to move (Kling, Liebman, & Katz, 2007). Although much debate about how to interpret the results, and whether the experimental design was adequate to find results given how few people opted to move or moved into neighborhoods that offered better opportunity (Aliprantis & Richter, 2012), has followed the MTO project, a few clear findings emerge. Adult mental health, for those who moved out of concentrated poverty (a small portion of the experimental group), improved significantly and those improvements have persisted over time (Kling et al., 2007).

Other studies have found similar, more robust, relationships between adult mental well-being and neighborhood effects. For instance, a ten-year longitudinal study of British civil servants examined neighborhood deprivation and social fragmentation, independent of individual socio-economic status, and found each associated with poorer mental functioning. The differences over time widened between those in more-compared-to-less fragmented neighborhoods as well as those in more-compared-to-less deprived neighborhoods, indicating a cumulative negative effect of deprivation and fragmentation (Stafford, Gimeno, & Marmot, 2008).

### 2.2 Children and Adolescents

As with adults, children's mental health and behavioral problems are also associated with neighborhood effects, most typically concentrated disadvantage (Caughy, Nettles, & O'Campo, 2008; Xue, Leventhal, Brooks-Gunn, & Earls, 2005). Children living in poverty were more likely than non-poor children to have a psychiatric disorder (Costello, Compton, Keeler, & Angold, 2003); psychological distress is higher among those who live in high poverty neighborhoods (Schulz et al., 2000); and those living in poverty have substantially worse physical health (Aber, Bennett, Conley, & Li, 1997). Poverty's consequences continue to effect children as they develop, with lowered education attainment, heightened risk of accidents, increased school drop-out, decreased IQ, increased risk for child maltreatment and neglect and increased behavior problems (Baydar, Brooks-Gunn, & Furstenberg, 1993; Duncan, Brooks-Gunn, & Klebanov, 1994; Furstenberg, Brooks-Gunn, & Morgan, 1987). When children moved from poverty to wealth in a natural experiment, after four years of affluence, psychiatric symptoms of previously poor children declined to match those children who had never been poor. This effect was strongest in the area of behavioral symptoms, e.g. conduct and oppositional disorder (Costello et al., 2003).

However, the evidence that changing neighborhoods improves child mental health is not always observed. For instance, children in the MTO had different outcomes of moving to less densely poor neighborhoods depending on gender and victimization, with girls' mental health improving but boys from vulnerable families experiencing a worsening mental health (Osypuk, Schmidt, et al., 2012; Osypuk, Tchetgen, et al., 2012). These findings suggest that neighborhoods in and of themselves are not the answer to mental health problems, but support the notion that they must be considered as influential variables when assessing risk and causation, and considered as part of the mechanism of both good and poor mental health outcomes.

Similar research explains that child problem behaviors may be explained, at least in part by measures of concentrated disadvantage. While controlling for individual family economic factors, the research indicates that neighborhood economic disadvantage has a significant impact on maladaptive behaviors. Thus, a child whose family's economic status is above the poverty line, but who lives in a neighborhood of concentrated disadvantage, is at heightened risk for behavior problems. Living in a disadvantaged neighborhood is an independent risk for behavior problems for children over time. This appears most significant during the transition from childhood to adolescence (Kalff et al., 2001; Schneiders et al., 2003).

Studies of twins raised apart found that variability in intelligence among children is related to the socioeconomic status of the family in which the twin was raised: for those raised in poverty, their poverty accounted for 60% of the variance in IQ scores while genes accounted for nearly none of the variance; for those raised in affluence, the results showed the opposite, with genes accounting for about 60% of variance (Turkheimer, Haley, Waldron, D'Onofrio, & Gottesman, 2003).

Other studies have found pre-frontal cortex deficits, manifested as executive dysfunction, related to socio-economic status (SES), although most of them have relied on global measures of SES and not sought to differentiate the influence of more closely defined neighborhood factors from SES. Nevertheless, impairments have been noted in cognitive flexibility, language performance and working memory associated with low SES (Kishiyama, Boyce, Jimenez, Perry, & Knight, 2009). Conversely, collective efficacy may underlie resilience in some children and may lower the incidence of mental illness (Xue et al., 2005).

## 3. Psychosis and Neighborhood

While depression and anxiety in relation to chaotic and fear-inducing neighborhood conditions makes a kind of simple conceptual sense (Cutrona, Wallace, & Wesner, 2006), the relationship between neighborhoods and psychosis is less intuitive. Nevertheless, many studies have now found strong associations between psychosis and neighborhood effects. A recent review found an increased incidence of schizophrenia across many countries, although primarily in Europe, for migrants (Cantor-Graae, 2007). A meta-analysis of 50 studies examining first and second generation migrants found a relative risk of 2.9 schizophrenia, with second generation immigrants having an RR of 4.5 and immigrants of color having an RR of 4.8 (Cantor-Graae & Selten, 2005).

Another review, which specifically examined urbanicity and neighborhood effects, found that both are associated with psychosis but that methodological problems in the reviewed studies undermine the ability to reach conclusions regarding neighborhood effects. Of the 44 studies reviewed, urbanicity was found to increase the risk of psychosis between two- and four-fold; and, despite methodological problems, many of the individual neighborhood effects studies found significant associations but were unable to reach broader conclusions (March et al., 2008).

Some of the current hypotheses regarding why the rates of psychosis are higher for some migrants include discrimination and social isolation. That is, that the social experience of migration, especially for migrants of color moving into countries which are predominantly white, contributes to the vulnerability and onset of psychosis. For instance, a study of psychosis in The Hague found the experience of racial/ethnic discrimination raised the incidence of psychosis, with high discrimination experiences leading to 4 incidence rate ratios compared to 1.2 incidence rate rations for very low discrimination exposure. Neighborhood measures included rates of long-term unemployment, income, poor quality of housing, and level of education. This study suggests that the perception of discrimination may contribute to increased risk for psychosis (Veling et al., 2007). Similarly, in a prospective study of the incidence of psychosis, immigrants living in neighborhoods where their own ethnic group comprised only a small percent of the population had higher incidence, suggesting that social isolation and the experience of exclusion may also play a role in the development of psychosis (Veling et al., 2008).

Moreover, a case-control study of first episode psychosis found that cases were more socially disadvantaged and isolated than controls. Six domains were included for assessing disadvantage and isolation: education, employment, living arrangements, housing, relationships and social networks. As the number of indicated risk factors rose, the incidence of psychosis rose as well. The initial risk for psychosis was generally similar for White British and Black Caribbean immigrants, but Black Caribbean immigrants had higher exposure to the indicated risk factors (Morgan et al., 2008).

One hypothesis, which needs further research, suggests that the higher rates of psychosis and the higher rates of child abuse that are associated with neighborhood effects may be related to each other. A recent study found a dose-response relationship in a prospective study between childhood trauma and the risk of psychosis, with an odds ratio for child abuse predicting psychosis of 7.3 percent (Janssen et al., 2004). Others have reported this association as well (Larkin & Morrison, 2006).

## 4. Childhood Physical /Sexual Abuse, Witnessing Violence and Neighborhoods

The long-term mental health consequences of childhood physical and sexual abuse and witnessing violence are well-established (Follette, Polusny, Bechtle, & Naugle, 1996; Herman, 1997; Kaplan et al., 1998; Malinoskyrummell & Hansen, 1993; Martinez & Richters, 1993; Maughan & Cicchetti, 2002; Roberts, O'Connor, Dunn, Golding, & Team, 2004; Van der Kolk, McFarlane, & Weisæth, 1996). Parental abuse of children has historically been viewed solely as a failure of parenting; not an unreasonable view, but one that may be too narrow when seeking to understand and prevent such abuse (Molnar, Buka, Brennan, Holton, & Earls, 2003). More recently, researchers have found considerable agreement across a range of studies that neighborhood disadvantage is also associated with child maltreatment. That is, neighborhoods have a significant effect on the incidence of childhood physical and sexual abuse even when controlling for individual and family differences (Coulton, Crampton, Irwin, Spilsbury, & Korbin, 2007). Abuse has been associated with economic and family resources, residential instability and geographic proximity to neighborhoods of concentrated disadvantage (Coulton, Korbin, Su, & Chow, 1995). Concentrated disadvantage and community violence significantly predicted parent to child aggression (Molnar et al., 2003). The measure of the concentrated disadvantage of the neighborhood typically refers to the percentage of residents below the poverty line, the percentage on family assistance, the percentage of female headed households, the percentage of unemployed, the percentage of children under 18, and the percentage of African Americans (Coulton, Korbin, & Su, 1999; Coulton et al., 1995; Molnar et al., 2003).

The statistical findings of the effect of neighborhoods on the incidence of abuse is significant but small because family factors interact with neighborhood factors. The interaction makes sense, because although the family is the actual mechanism of the maltreatment, neighborhood-level factors such as economic and family resources, residential instability, household make-up, and geographic proximity to concentrated poverty areas are all associated with the occurrence of maltreatment. Thus, while this research indicates that neighborhoods do not primarily *cause* child maltreatment, neighborhood factors are part of the understanding of how and when and why child maltreatment occurs. Neighborhoods with the highest maltreatment rates were those with high combinations of poverty, unemployment, racial segregation, abandoned housing, population loss, lack of child care, few elderly residents, and which border other neighborhoods with high density poverty. These structural factors explain a significant part of the statistical variance in maltreatment across neighborhoods (Coulton et al., 1995).

Moreover, witnessing community violence has been shown to be associated with both behavioral and psychological problems in youth. The individual effects of exposure to community violence (depression, withdrawal, dissociative coping, aggression, substance abuse, stress, post-traumatic stress disorder and other physiological deficits) are significant (Buka, Stichick, Birdthistle, & Earls, 2001; Salzinger, Feldman, Stockhammer, & Hood, 2002). Witnessing community violence is related to child maltreatment and poor outcomes as both the “severity of neglect and victimization by violence in the community are significant predictors of children's functioning” (p. 246) (Lynch & Cicchetti, 1998). The neighborhood can create increased risk for both exposure to violence and for physical and sexual abuse within the family (Garbarino & Sherman, 1980).

A community survey found that where there were lower than expected rates of child abuse there was higher reported satisfaction with neighborhoods (Garbarino & Sherman, 1980). They suggested that neighborhood, and community member perception of the neighborhood, was an important factor in child maltreatment. Similarly, a neighborhood with a high degree of collective efficacy may in fact provide a protective effect against child abuse. Meaning, where the neighborhood has strong social cohesion, that cohesion may provide a protection for children against the violence of a parent (Silk, Sessa, Morris, Steinberg, & Avenevoli, 2004). Neighborhoods have been associated with resiliency as well, meaning, neighborhood advantage may assist abused children in more quickly overcoming and coping with the abuse (DuMont, Widom, & Czaja, 2007).

## 5. Neurotoxicant Exposures

Exposure to neurotoxicants, in particular pesticides, metals and solvents, is also a pervasive neighborhood-level problem that has significant effects on developmental course (Landrigan et al., 1999). For instance, exposure to DDT *in utero* has been found to cause significant neuro-developmental delays (Eskenazi et al., 2006). Similarly, childhood exposures to pesticides and flame retardants have been associated with persistent neuro-developmental delays (Eskenazi, Bradman, & Castorina, 1999; Eskenazi et al., 2013). The neighborhood level issues result from the unequal distribution of environmental hazards such that they disproportionately expose some people more than others (Cole & Foster, 2001). The effect of lead, for instance, has clearly been shown to be unequally distributed by both race and class, as well as being related to a host of negative health and mental health outcomes (Hu, Shih, Rothenberg, & Schwartz, 2007). The likelihood and extent of exposure to neurotoxicants is dependent, at least in part, on neighborhood factors such as segregation and density of poverty and public policy related to the zoning of hazards and housing; these factors in turn shape the developmental course of those who live in neighborhoods with high levels of neurotoxic agents (Bullard, Johnson, & Torres, 2000).

Researchers have sometimes viewed neurotoxicant exposure as a confound of poverty. This appears to be an inaccurate inference, however, and may lead to a false attribution of effects (Bellinger, 2008). Instead, Bellinger has argued that lead exposure is an effect modification with potential direct influence on dose-response relationships, and therefore on health outcomes (Bellinger, 2000). Therefore, rather than “controlling” for context when assessing the effect of neurotoxicant exposure on health and behavior, this research suggests a more careful consideration of the effect modification of neighborhood level effects when considering the outcomes of exposure.

Both high exposures (including poisoning) and chronic, low-level exposures are associated with a host of negative health and mental health effects. A wealth of research indicates that chronic pesticide exposure is associated with decreased cognitive, psychomotor and psychiatric functioning (Kamel & Hoppin, 2004). These changes can persist throughout the life time of the exposed person and can be dramatic (Ecobichon & Joy, 1994; Feldman, 1999).

For children, from *in utero* to adolescence, the human body is less able to physiologically eliminate pesticides compared to adults. The absorption in children is more than 70% compared to absorption in adults of 30% of many chemical agents. In addition, because the central nervous system is developing during the course of adolescence and vulnerable to toxic mutation, exposure prior to and during adolescence alters the development and functioning of the brain to a greater degree (Rice & Barone, 2000).

Perhaps more than any other agent, the neurocognitive effects of lead have been studied extensively. Declines in IQ scores have been demonstrated over many years (Canfield, Henderson, et al., 2003; Lanphear et al., 2005); reduction in brain volume, specifically in frontal gray matter and the anterior cingulate cortex, has more recently been shown (Cecil et al., 2008); behavioral problems, including arrests, as well as neuropsychological impairments such as spatial attention, executive functioning, attention, working memory and learning (Canfield, Kreher, Cornwell, & Henderson, 2003; Surkan et al., 2007; Wright et al., 2008). Lead exposure has also been associated with depression, anxiety, irritability and anger (Shih, Hu, Weisskopf, & Schwartz, 2007).

In adults, the cumulative exposure to lead is associated with increased neurocognitive decline. The most significant association between bone-lead level and cognitive decline was found in total cognitive score, spatial ability, learning and memory, and in executive functioning over time compared to controls (Khalil et al., 2009). The consequences of cumulative life-time lead exposure are exacerbated by neighborhood level psychosocial hazards. The combination resulted in diminished cognitive functioning in executive function and language areas for adults. The lead exposure-cognitive impairment demonstrated dose-response characteristics and has a plausible biological mechanism (Glass et al., 2009).

Pesticides have also been shown to significantly increase the risk for mental illness based on both chronic exposure and poisoning. While pesticide poisoning increased the risk of depression by an odds ratio of 2.57%, chronic exposure increased the odds ration by 1.54 percent (Beseler et al., 2008). Pesticides (primarily organophosphates and organochlorines) are also associated with a host of symptoms that may be short- or long-term in duration: irritability, depression, anxiety, mood lability, agitation, memory impairment, confusion, hallucinations, academic deficits, hyperactivity, poor concentration, paranoia, dissociation and somatic complaints (Brown, 2002).

Finally, environmental deprivation may have a potentiating effect on neurotoxicant exposure deficits. Put more positively, environmental enrichment can serve as a tool to assist exposed children in being resilient. Heightened stress (such as exposure to violence or negative neighborhood factors) and heightened maternal stress also appear to negatively interact with neurotoxic exposure (Cory-Slechta, Virgolini, Thiruchelvam, Weston, & Bauter, 2004; Weiss & Bellinger, 2006). In this way, a mechanism by which neurotoxic exposure and the other neighborhood level factors discussed can be understood to relate to worsened mental health outcomes.

## 6. Criminal Behavior

As discussed above, immigration studies first found a “race” effect for psychosis, but later research suggests that the perception of discrimination and migration status itself, as well as neighborhood effects, may be the more important mechanisms in understanding the observed findings. This is not to argue for a more limited view of the importance of race and class, but rather for a more precise measurement -- and as a result of so doing, to shift the individual-level markers of race and class back to the structural-level of their operation and effect and to understand for what they stand as proxies (Manly, 2006; Manly & Echemendia, 2007).

No area of research more so than studies of criminal behavior has been confounded by issues of race and class. Having reviewed some of the neighborhood effects literature on mental health, the question arises as to how neighborhood effects may relate to criminal behavior. Are the same factors observed in the health and mental health research found in the research on criminal behavior?

Recently, the role of race and class in the perception of neighborhood disorder has attracted attention, with some research suggesting that the racial and class make-up of a neighborhood shapes people's perceptions of disorder more than trash, graffiti or broken windows (Franzini, Caughy, Nettles, & O'Campo, 2008). This research runs directly counter to the “broken window” theory of crime, first posited in 1982 (Wilson & Kelling, 2011), which suggested that public disorder of any sort leads to criminal behavior (Kelling & Coles, 1996). This theory argues that the unrepaired window leads to the breakdown of community social control, and therefore increased crime. The idea that race and class perceptions, rather than disorder, effect residents' view of neighborhoods undermines the proposition that disorder is causative of violence.

However, when the question of race and crime are analyzed directly, the results indicate that neighborhood disadvantage rather than race explains significantly more variation in crime rates. Individual differences (family poverty status, IQ, and impulsivity) accounted for about 6% of variance between white and African-American crime, whereas neighborhood disadvantage (including racial segregation) explained 60% of the difference (Sampson, Morenoff, & Raudenbush, 2005). Further, variation between neighborhoods is more significant to understanding crime than race, meaning that crime rates for whites and African-Americans are almost identical when controlling for neighborhood level differences (Peterson, Krivo, & Hagan, 2006). These findings echo those of McNulty and Bellair who reported that differences between neighborhoods, rather than people, explained criminal youth violence (McNulty & Bellair, 2003).

In fact, in an analysis of Chicago neighborhoods and homicide, researchers found that spatial proximity to violence, collective efficacy and measures of affluence/resource inequality were the most significant predictors of variations in homicide rates (Morenoff, Sampson, & Raudenbush, 2001). This research challenges the view that minor disorder leads to major crime, finding instead that low collective efficacy neighborhoods tend to be higher in both disorder and criminal activity (Sampson & Raudenbush, 1999; Sampson, Raudenbush, & Earls, 1997a). Most interestingly, this research has shown that where collective efficacy is high, that is, where neighbors have shared expectations and the neighborhood has a strong sense of cohesion, even where poverty is concentrated, crime is low; and in a analysis of homicides, the findings are even stronger that the combination of collective efficacy and measures of inequality are exceptionally strong predictors of homicides (Morenoff et al., 2001).

Relatedly, research has shown that impulsive boys were at greater risk for juvenile offending if they lived in densely poor neighborhoods compared to impulsive boys who lived in better neighborhoods (Lynam et al., 2000). As suggested by this research, the interaction between individual factors, such as mental illness, and neighborhood effects, such as dense poverty or collective efficacy, suggests that context is critical to understanding behavior and outcomes of concern such as criminal offending.

## 7. Neighborhood Effects Mechanisms and Implications

The research reviewed here on crime and mental illness suggests that neighborhood effects must be considered if the determinants of behavior and functioning are to be understood. Neighborhoods are defined by physical space, shared social norms and expectations, social networks, and institutional structures, and neighborhoods do appear to play a role in the mechanism by which some people develop mental illnesses, and behavioral and functional impairments. Sampson (2008) argues for understanding neighborhood effects mechanisms on individual behavior in two ways: first, in the situational context of life-course in a place; and second, in the developmental and enduring effects that neighborhoods exercise on early life course that may persist throughout the life of the individual regardless of neighborhood stability or individual mobility (Sampson, 2008). Certainly the known long-term effects of childhood exposure to violence, childhood exposure to neurotoxicants and the onset and course of many serious mental illnesses, support the notion of studying neighborhood effects in this way. That the effects long outlast changed circumstances supports the concept of examining both context and developmental course.

The literature on neighborhood effects would appear to point to collective efficacy and concentrated disadvantage as the ways in which individuals are shaped by neighborhoods. Collective efficacy, which refers to the level of mutual trust and cohesion among residents and their willingness to work toward the common good is related to the structural characteristics of the neighborhood. Collective efficacy includes a shared willingness and capacity for people in a neighborhood to intervene informally (exercise informal social control) in neighborhood activities to promote social good. Research has shown that where collective efficacy is high, that is, where neighbors have shared expectations and the neighborhood has a strong sense of cohesion, even where poverty is concentrated, crime is low (Sampson et al., 1997a).

Alternatively, in neighborhoods in which collective efficacy is low, residents may feel isolated and have little belief in the neighborhood's capacity to improve negative situations, such as drug dealing or crime. In such neighborhoods residents are less willing to enforce conventional behaviors or provide control for inappropriate activities that occur (Earls & Carlson, 2001; Sampson, Morenoff, & Earls, 1999).

Neighborhood effects research has found that concentrated disadvantage and residential instability explain 70% of neighborhood variation in how willing people are to help their neighbors, intervene on their behalf or protect other people's children (Sampson, Raudenbush, & Earls, 1997b).

A recent summary of neighborhood research finds that

[T]he evidence is solid on the ecological differentiation of American cities along socio-economic and racial lines, which in turn corresponds to the spatial differentiation of neighborhoods by multiple child, adolescent, and adult behaviors. These conditions are interrelated and appear to vary in systematic and theoretically meaningful ways with hypothesized social mechanisms such as informal social control, trust, institutional resources and routines, peer-group delinquency, and perceived disorder. An important take-away of our assessment is that these and other neighborhood-level mechanisms can be measured reliably with survey, observational, and archival approaches.

(p. 473) (Sampson, Morenoff, & Gannon-Rowley, 2002).

Neighborhood processes can and should be treated as ecological or collective phenomena rather than as individual-level perceptions or traits. Collective efficacy is a measure of informal social control and mutual dependence, where people believe that members of their community will assist them when they are in need. Nevertheless, collective efficacy has an independent effect on both contextual and life-course development of mental illness and criminal behavior. This effect is significant enough that it should not be overlooked or studied by proxy measures.

Similarly, concentrated disadvantage, rather than vague notions of socioeconomic status or other proxy markers, have demonstrated robust impact on the context and life-course of individuals as indicated when looking at mental illness and criminal behavior as outcomes. Although often studied through proxy measures that fail to adequately address how and why concentrated disadvantage operates, significant findings point to concentrated disadvantage having life-course effects (Sampson, 2008).

Perhaps equally significant in this research are the questions that are raised about how we understand race and class. Both have been used variously as proxy measures to the detriment of more nuanced understandings. Each needs to be deconstructed if we are to explore the actual mechanisms of a host of behavioral, psychological and health outcomes.

The evidence that the proxy measures of race and class have clear health and behavior consequences is not undermined by seeking a better understanding of what is meant when those categories are used to group people. Residential segregation is the clearest means by which these structural factors shape people's lives by defining their access to, and the quality of, medical and social services, employment, education, food, mobility, environmental hazards and a host of disadvantage (Acevedo-Garcia, Osypuk, McArdle, & Williams, 2008).

Racial segregation in housing not only affects individuals but also acts as a social and neighborhood structural barrier. A study of urban Atlanta found substantial race-based discrimination in housing and work, including a spatial mismatch (poor people who need entry level jobs are unable to live in proximity to those jobs) and housing segregation (inability to move based on race), which resulted in a concentration of poor people into densely poor areas (Sjoquist, 2000). These neighborhood factors shape the lives of people by narrowing their options and teaching mean lessons about what it means to be poor or a person of color (W. J. Wilson, 1996). Nationally, African Americans among all racial groups are most physically segregated from jobs (Stoll & Raphael, 2002). In terms of mental health care, in 2001, the Surgeon General of the United States issued a report finding serious disparities in mental health care and treatment for people of color. The report found that people of color are less likely than whites to receive services and more likely to receive poor quality services when they do (Health and Human Services, 2001). In addition, perceptions of discrimination are associated with poorer mental and physical health (Williams, Neighbors, & Jackson, 2003).

Further, consideration of neighborhood disadvantage accounts for some of the race/ethnic and socioeconomic position differences in health, particularly in hypertension. When controlling for neighborhood effects, a significant amount of the statistical difference in the incidence of hypertension that appears based on racial/ethnic status goes away (Morenoff et al., 2007).

When looking at violence in patients recently discharged from a psychiatric hospital, researchers found that, after controlling for individual factors (such as age, diagnosis, prior arrests), the concentrated disadvantage of the neighborhood into which the patient was discharged was predictive of future violence. That is, the risk that a patient discharged from locked facility would engage in future violence increased 2.7 times if the person was discharged into a neighborhood of concentrated disadvantage compared to a less disadvantaged one (Silver, 2000; Silver, Mulvey, & Monahan, 1999). But they also found that “the significant association between African-American racial status and violence was completely eliminated when neighborhood disadvantage was controlled” (p. 405) (Silver, 2001).

In a study of stereotype threat, researchers found that simply asking African-Americans to record their race before a test significantly lowered test performance compared to whites and compared to African-American controls (Steele & Aronson, 1995). Whatever else this research says, it provides some insight into how structural barriers to equality, perhaps ones that are not context-driven but which shaped the developmental and experiential life-course of the test subjects, act on individuals. Simply examining how these subjects scored on tests would lead to false conclusions and useless intervention strategies. Similarly, research that relies on race and class as proxies about crime, violence or mental illness are missing critical information which might re-shape the understanding of cause and course of behavior and illness. Instead, neighborhood effects, in their specific mechanisms of action, can assist in deconstructing those proxy measures such that a more accurate and meaningful understanding can be sought.

How we understand race and class effects should be broadened to look at both the context effect of discrimination, isolation and perception, as well as the long-term effect on mental health and criminal behavior. When Sampson argued for this dual understanding, it was in order that the research begin to take into account both mechanisms (Sampson, 2008). Neighborhood effects have both a contextual consequence as well as a long-term developmental consequence, and the outcomes discussed here also demonstrate evidence of such influences. Yet, we often ignore the developmental and course aspects of both, which means we are missing important prevention and intervention evidence as well as misconstruing the results of the data. How we understand neighborhood effects, both collective efficacy and concentrated disadvantage, should also be helping us understand the context and life course of individual behavior and illness.

This should also affect how forensic assessment is conducted. Competent forensic neurobehavioral assessment requires a thorough multigenerational social history, along with the integration of information obtained from multiple sources, across a number of disciplinary approaches, each of which assess different aspects of behavior and functioning (Woods, Freedman, & Greenspan, 2012). It is no longer adequate to consider the individual out of context or to seek to explain behavior and functioning without regard to the causal effects of structural forces. Forensic assessment, in our view, must include an assessment of those structural forces to be meaningful and valid.

This means that popular forensic views about individuals characteristics and actions will need to be replaced with scientifically reliable and valid evidence of the dynamic forces which shape the life course. Behavior and functioning will need to assessed from the viewpoint of the causal forces that shape an individual from place in which they are born, to the cultural and ethnic biases they have faced, to the opportunities available or denied. Competent forensic assessment should consider the ways in which neighborhood effects, as one example of the structural forces demonstrated to affect behavior and functioning in mental illness and crime, alter the life trajectory of the individual.
